# Chronic and Residual Effects of a Two-Week Foam Rolling Intervention on Ankle Flexibility and Dynamic Balance

**DOI:** 10.3389/fspor.2022.799985

**Published:** 2022-02-15

**Authors:** Thomas Christoph Seever, Joel Mason, Astrid Zech

**Affiliations:** Department of Human Movement Science and Exercise Physiology, Institute of Sports Science, University of Jena, Jena, Germany

**Keywords:** roller-massage, foam rolling, range of motion, ankle dorsiflexion, balance

## Abstract

**Background:**

Foam rolling has been shown to acutely improve joint range of motion (ROM). However, limited knowledge exists on the chronic and residual effects. The primary purpose of this study was to examine the chronic and residual effects of a 2-week roller–massager intervention on ankle dorsiflexion ROM and dynamic balance.

**Methods:**

Forty-two participants (24.3 ± 2.5 years, 33 males, 9 females) were randomly assigned to either roller-massage (RM) or control group (= no intervention). Ankle ROM was assessed with the weight-bearing lunge test (WBLT) and dynamic balance with the Y-Balance test for both limbs. The RM group was instructed to roll their calf muscles for three sets of 60 s per leg on 6 days a week over 2 weeks. Acute effects were measured during baseline testing for dorsiflexion ROM and dynamic balance immediately after foam rolling. Chronic and residual effects were measured 1 day and 7 days after the intervention period. Multivariate ANOVA was performed for *post-hoc* comparisons to determine acute, chronic, and residual effects.

**Results:**

Significant acute and chronic foam rolling effects (*p* <0.05) were found for ankle dorsiflexion ROM. The chronic increase in ROM slightly decreased 7 days post-intervention but remained significantly above baseline (*p* < 0.05). Regarding dynamic balance, there were no acute but chronic (*p* < 0.05) and residual (*p* < 0.05) effects.

**Conclusion:**

Using a roller–massager for a 2-week period chronically increases ROM and dynamic balance. These increases are still significant 7 days post-intervention emphasizing the sustainability of foam rolling effects.

## Introduction

Restricted ankle dorsiflexion range of motion (ROM) has been associated with altered lower extremity movement patterns (Rabin et al., 2016; Lima et al., 2018), with potential to influence sports performance and place undue strain on surrounding connective tissues which may ultimately lead to injury (Hewett et al., 2005; Boling et al., 2009). It is thus not surprising that deficits in dorsiflexion ROM have also been linked to decreased postural control (Hoch et al., 2011; Basnett et al., 2013) and increased risk of chronic ankle instability (Hertel and Corbett, 2019), Achilles tendinopathy (Rabin et al., 2014), patellofemoral pain (Piva et al., 2005), anterior cruciate ligament injury (Fong et al., 2011), and hamstring injury (Gabbe et al., 2006). Considering these detrimental effects on functional performance and injury status, the improvement of ankle ROM may be of high practical relevance for recreationally active and sporting populations.

Muscle stretching is arguably the most common and effective strategy to enhance joint ROM both acutely (Behm et al., 2016) and chronically (Thomas et al., 2018). Despite its efficacy, there is growing interest in foam rolling as an alternative treatment method. This increased interest is reflected in the recent proliferation of systematic reviews assessing the effects of foam rolling on various functional outcomes (Hughes and Ramer, 2019; Wiewelhove et al., 2019; Hendricks et al., 2020; Skinner et al., 2020; Wilke et al., 2020). Besides improved recovery (Kalén et al., 2017), physical performance (Halperin et al., 2014; Peacock et al., 2014), and decreased pain perception (Aboodarda et al., 2015), it has been established that foam rolling can induce acute ROM changes comparable to those of muscle stretching (Wilke et al., 2020). However, there are mixed and limited findings regarding the chronic ROM changes following foam rolling interventions.

To date, only three studies have examined the long-term effectiveness of foam rolling on ankle dorsiflexion ROM (Aune et al., 2019; Smith et al., 2019; Kiyono et al., 2020). Of these studies, Kiyono et al. (2020) and Smith et al. (2019) observed significant improvements after a 5- and 6-week period of calf muscle foam rolling, respectively. Aune et al. (2019), by contrast, did not observe significant training-induced changes following a 4-week intervention of daily foam rolling. One possible explanation for the inconsistency between these three studies might be the difference in training variables, given that all aforementioned studies prescribed different intervention set, repetition, and frequencies. However, this fails to explain the different results between Junker and Stöggl (2015) and Hodgson et al. (2018) who both investigated the chronic effects of a 4-week foam rolling intervention on hamstring flexibility. Despite similar training protocols regarding training duration, frequency, and volume, only Junker and Stöggl (2015) discovered significant improvements in ROM.

Similarly sparse and inconclusive is the evidence relating to the effects of foam rolling on static and dynamic balance. Of the four studies that have explored the impact upon balance (Halperin et al., 2014; Grabow et al., 2017; Lee et al., 2018; Junker and Stöggl, 2019), only one reported significant improvements in dynamic postural control immediately after foam rolling the quadriceps and hamstrings (Lee et al., 2018). Neither Halperin et al. (2014) nor Grabow et al. (2017) found significant increases in static balance performance directly after rolling the calf muscle and the plantar sole, respectively. Finally, no training-induced changes in dynamic balance were observed by Junker and Stöggl (2019) following an 8-week foam rolling training intervention for the lower body.

Given these mixed findings, research into foam rolling is indeed still in its infancy (Hendricks et al., 2020). There are currently no dosage recommendations to achieve optimal long-term effects for performance or therapeutical effects. Further work is needed especially to determine the long-term (chronic) effectiveness of foam rolling on ROM, and to elucidate its effects on balance, both of which are important determinants of functional performance and injury prevention. This information may help athletes and coaches/athletic trainers to better implement foam rolling interventions into the training routine. Therefore, the primary aim of this study was to examine the chronic and residual effects of a 2-week roller–massager intervention on ankle dorsiflexion ROM and dynamic balance, with a secondary aim to explore the acute effects of roller-massaging on the same outcome measures. It was hypothesized that (1) the 2-week training program would increase ROM which, in turn, would lead to improvements in dynamic balance, (2) potential chronic improvements would remain above baseline levels 7 days post-intervention, and (3) one bout of roller-massaging would cause acute increases in ROM which would be accompanied by improvements in balance performance.

## Materials and Methods

### Participants

A total of 42 participants were recruited for this study. Inclusion criteria were an age between 18 and 35 years and regular physical activity (two times per week or more). Exclusion criteria were a baseline flexibility ≥18 cm in the weight-bearing lunge test (WBLT), recent injury to the lower body that could have affected the performance on the WBLT and the Y-Balance test, and regular foam rolling of the calf muscles (≥once a week) prior to the investigation. The study was carried out in accordance with the medical research guidelines of the Helsinki Declaration and ethical approval was granted by the local ethics committee. Written consent was obtained from all participants.

A power calculation for a repeated measures ANOVA (within-between interaction) was performed *a priori* revealing a required sample size of 40. This calculation was based on the following input parameters: effect size of 0.3, alpha level of 0.05, power level of 0.95, two groups, and two measurements. To account for possible dropouts, 42 participants were included. Effect size calculation was based on data of own studies using the WBLT and Y-Balance test (John et al., 2019; Rahlf et al., 2020).

### Experimental Approach

**Figure 1:**
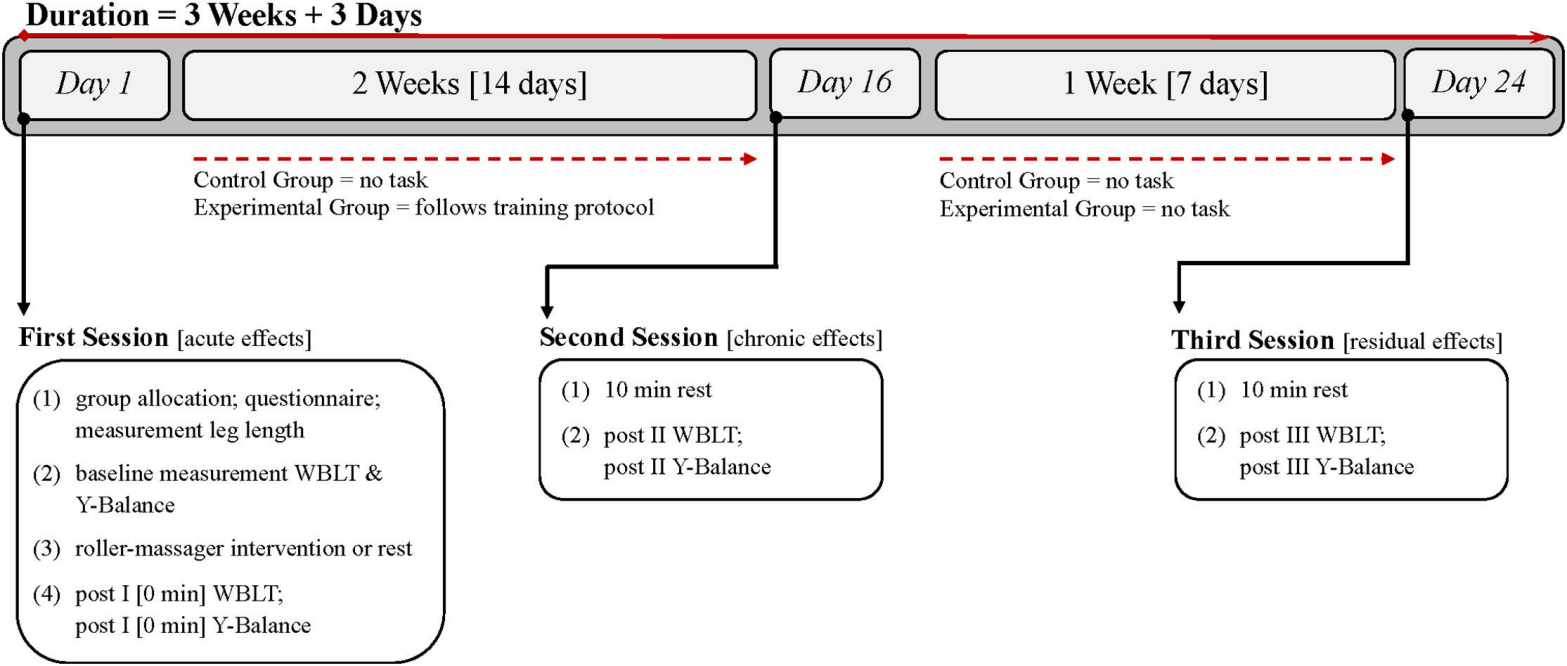
Timeline of measurements and intervention.

A randomized controlled between-subject design was used to explore the acute, chronic, and residual effects of calf muscle foam rolling on ankle dorsiflexion and dynamic balance. Throughout the 24-day study period, data were gathered on three separate occasions. The appointments were arranged at 12 p.m. or later but not earlier. An effort was made to assure that all three assessments every individual was to participate in were undertaken at approximately the same time of day in the laboratory. The participants were instructed to refrain from strenuous physical exercise of the lower body for at least 24 h prior to each appointment. The timeline of the entire study procedure is visualized in Figure 1.

Before the first assessments on *Day 1*, a simple randomization procedure was used for group assignment by having the participants draw a small piece of paper showing either 0 (= passive control group) or 1 (= experimental group). Demographic data were then collected with a questionnaire, and limb length was measured. After test familiarization, baseline assessments for ankle dorsiflexion (WBLT) and dynamic balance (Y-Balance test) were undertaken. Subsequently, the roller–massager intervention was explained to the experimental group by the researcher. The rolling technique was then practiced, and the treatment began once the participant was familiar with the technique. This process took 12 min on average. Participants in the control group spend 12 min lying in supine position on a yoga mat. Immediately after the 12 min, post I WBLT and Y-Balance test measurements were performed for both groups to measure acute effects.

After the first test session, the experimental group was instructed to follow the unsupervised foam-rolling training protocol for 2 weeks, starting on the next day. Controls were asked not to change their exercise habits for the duration of the measurement period. On *Day 16*, 1 day after the last foam rolling intervention, both groups attended the laboratory for a second time (= post II) in order to determine chronic effects. Upon arriving, participants were first instructed to lie down in supine position for 10 min to minimize any warm-up effect. Afterwards, the WBLT and the Y-Balance test were performed.

The third week was treatment-free for both groups. The last assessment (= post III) was on *Day 24* to examine residual effects of the foam rolling intervention. The procedure of the third session was identical to the second one.

### Roller–Massager Protocol

The tool used for the investigation was a customized roller–massager (3.5 cm in diameter) made from beech wood with a smooth surface structure. It consists of two handles (each 10 cm in length) and one center piece (20 cm in length) which were attached to one another by a round metal bar (0.8 cm in diameter). To minimize friction and thus facilitate a smooth rolling motion, the metal bar was lubricated prior to assembly. Two stainless steel washers were added as a spacer between the center piece and the two handles.

**Figure 2:**
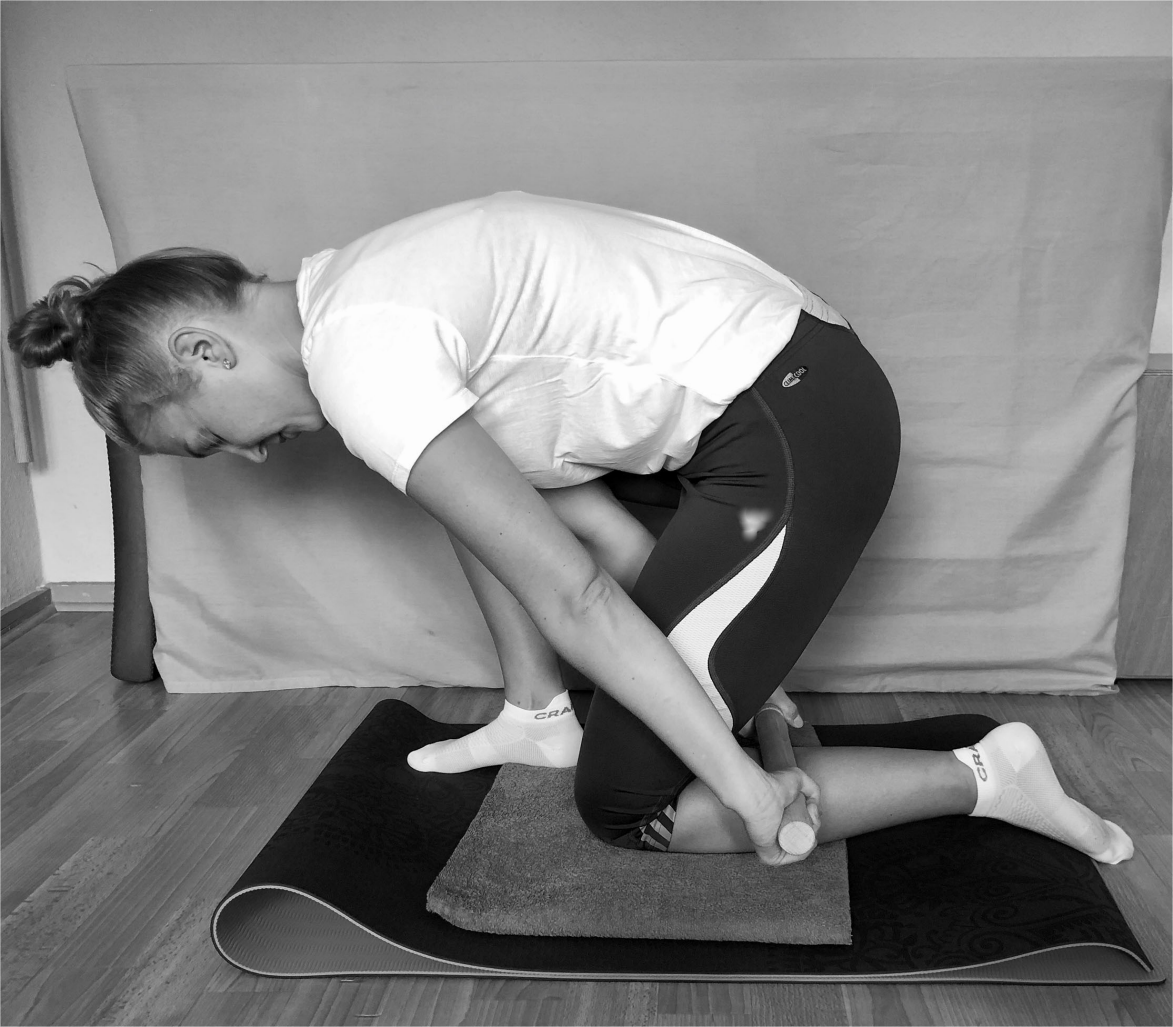
Roller massage technique.

The rolling was performed bilaterally by each participant in a half-kneeling position (Figure 2). The leg undergoing the treatment was on the floor with a cushioned support under the knee. The contralateral leg was squatting to provide enough space for the rolling motion. The roller–massager was then applied to the triceps surae moving from the hollow of the knee to the Achilles tendon and back (= one repetition). The same procedure was then performed on the other leg.

The foam rolling protocol used for the acute effects was adopted from Behm et al. (2020) and comprised three sets of 60 s for each leg. Individuals were required to switch leg sides after each set with no obligatory rest in between, apply as much pressure as possible, and roll at the speed of 60 beats per min (one stroke per beat). One repetition consisted of four downward and four upward strokes. The experimental group performed the rolling with the same protocol in the subsequent 2-weeks on 6 days per week. The timing of the weekly rest day could be chosen freely. Foam rolling was mandatory at the very first and the last day of the intervention.

### Weight-Bearing Lunge Test

The WBLT was used to assess ankle dorsiflexion ROM. The WBLT has been used due to sensitivity to detect restrictions or improvements in ankle ROM (Hall and Docherty, 2017). For the test a good to excellent reliability (ICC between 0.65 and 0.99) is reported (Powden et al., 2015). The general set-up used in this study is in accordance with previous investigations (Halperin et al., 2014; Škarabot et al., 2015; Kelly and Beardsley, 2016). Participants were instructed to stand in front of a 90 cm tall, wooden box in an upright lunge position without shoes. Their hands were allowed to rest on top of the box. The big toe of the leading leg had to be aligned with the 10 cm mark (= starting point) of the tape measure, which was attached to the floor at an angle of 90° to the box. If the second toe was longer than the big toe, it was used for the alignment instead. The anterior knee was then moved in a straight line toward the wooden wall. If this movement was slow and controlled, and the contact to the wall could be maintained for at least one second without any heel lift of the assessed limb, the trial was valid. Heel elevation was controlled with a resistance band (TheraBand®, resistance level yellow) that was placed underneath the heel of the assessed ankle and held under tension by a 5 kg weight plate. Depending on the outcome of each trial, the foot was gradually moved toward or away from the wall by 0.5 cm until the maximum ROM of the ankle joint was achieved. The number of trials was unlimited, and the results were measured to the nearest 0.5 cm. Ankle ROM was determined for both limbs.

### Y-Balance Test

The Y-Balance test was used to determine dynamic postural control. The test has been widely used in athletes and is considered both reliable (ICC between 0.73 and 1.00) (Powden et al., 2019) and valid (Gribble et al., 2012) in adult individuals. To form the anterior, posteromedial, and posterolateral reach, three tape measures were attached to the floor in the shape of a reversed Y (Plisky et al., 2006). Participants performed six practice and three recorded trials on both legs in each of the three excursion directions. Regardless of reach direction, the participant had to position their big toe of the stance leg behind a straight line at the center of the Y (Stiffler et al., 2015). A trial was accepted if the participant was able to maintain postural control, keep both hands on their hips, touch the floor gently with the reach foot without putting weight onto it, and return the reach foot to the starting position in a slow and controlled manner. Heel elevation was again controlled with a resistance band but only during the anterior reach.

The data were recorded to the nearest 0.5 cm. To normalize the reach distance, the maximum reach of each direction was divided by the mean value of the participant's limb length and multiplied by 100 (Gribble et al., 2012). To calculate the normalized composite score, maximum anterior, posteromedial, and posterolateral reach were added up, divided by the three-fold mean value of the participant's limb length, and multiplied by 100. Limb length was measured bilaterally from the inferior part of the anterior superior iliac spine to the tip of the medial malleolus.

### Statistical Analysis

Statistics were performed with SPSS (version 27.0.0.0). For each participant (*n* = 42) data of both legs (*n* = 84) were used for statistics. Baseline data of continuous variables were presented as mean and standard deviation. Baseline differences between groups were tested with the *t*-test for two independent groups and Pearson's chi-squared test. Time as well as group by time interaction effects for changes over the four time points (baseline, post-tests I, II, and III) were tested with two-factor repeated-measures ANOVA. Greenhouse-Geisser corrections were applied when sphericity was not met according to Mauchly's Test of Sphericity. Levene's test was used to examine homogeneity of variance. To account for baseline differences between groups, a multivariate ANOVA was used for *post-hoc* comparisons. Accordingly, the dependent variables were changes in WBLT and Y-Balance scores from baseline to post I (acute effects), baseline to post II (chronic effects), and baseline to post III (residual effects). The independent variable was group (control and treatment). Statistical significance was set to an alpha level of < 0.05.

## Results

**Figure 3:**
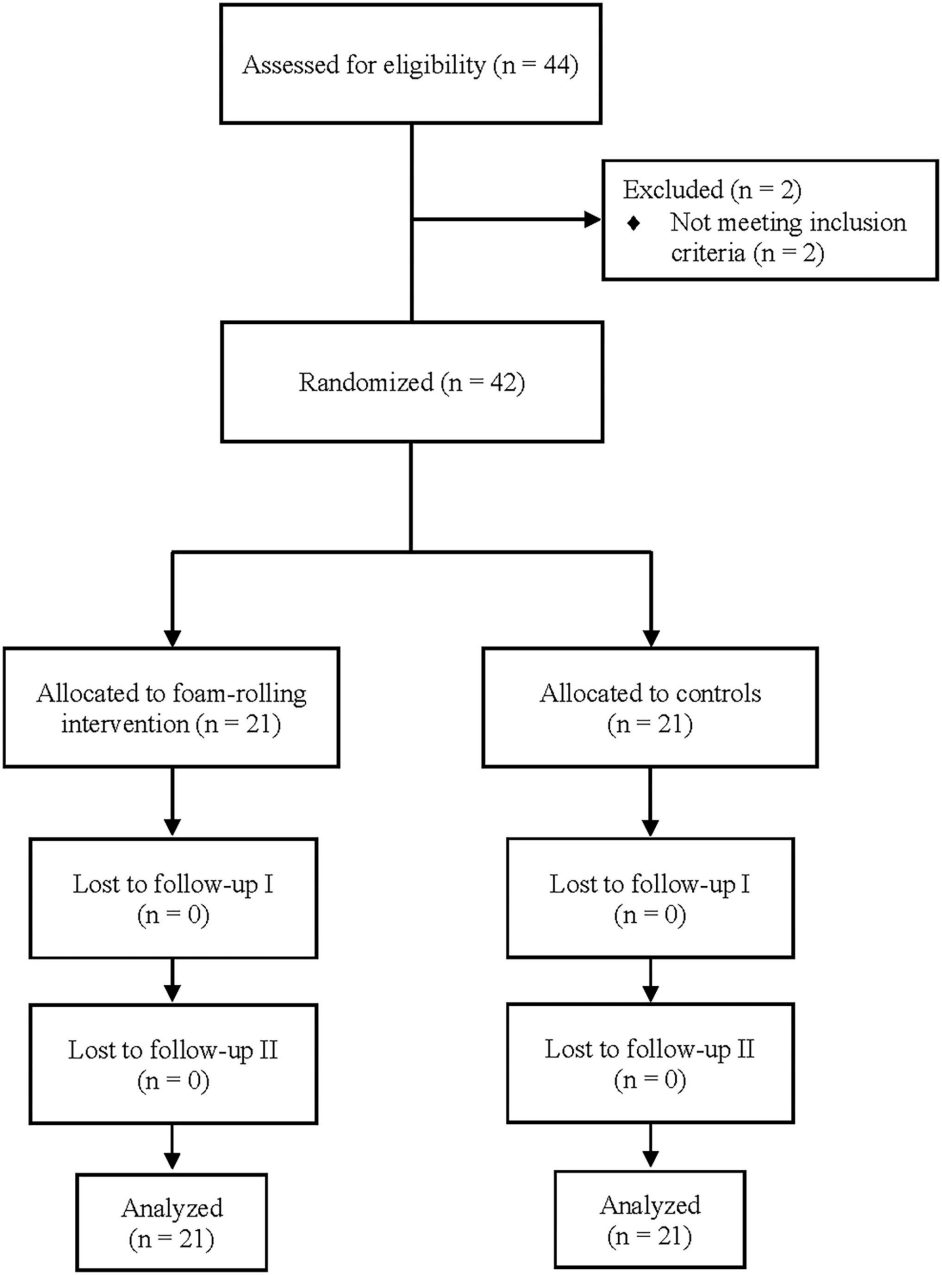
Study flow chart.

A total of 44 participants were screened for eligibility. Two of these were excluded, as they did not meet the inclusion criteria. The remaining 42 participants were randomized and allocated to either roller-massage (RM) (*n* = 21) or no RM (*n* = 21). Twenty-four participants (treatment: *n* = 11, controls: *n* = 13) reported previous injury to the lower limb of which ankle sprains were mentioned most often. In general, the physical activities were identical between both groups including running (treatment group: *n* = 17, controls: *n* = 15), cycling (both *n* = 9), strength training (treatment: *n* = 9, controls: n =5), soccer (treatment: *n* = 6, controls: *n* = 4), volleyball (treatment: *n* = 3, controls: *n* = 2), and swimming (treatment: *n* = 9, controls: *n* = 5). Throughout the study process no dropouts were recorded. Thus, 21 participants were included in the final analysis for each condition. The flow chart of the study process is presented in Figure 3.

Participant characteristics and baseline data of the WBLT and the Y-Balance test are shown in Table 1. No significant differences between groups were observed for the WBLT at baseline. However, all Y-Balance pre-test scores were significantly (*p* < 0.05) different across groups.

**Table 1:** Participant characteristics and baseline values of WBLT and Y-Balance test.

	**All**	**Treatment**	**Controls**	**Group differences (*P*-value)^a^**	***T***
**Sample size**	42	21	21		
**Age** (years)	24.3 ± 2.5	24.2 ± 2.6	24.4 ± 2.4	0.724	−0.354
**Weight** (kg)	74.3 ± 10.9	72.1 ± 10.6	76.6 ± 11.1	0.056	−1.937
**Height** (cm)	177.6 ± 7.4	176.4 ± 7.3	178.8 ± 7.6	0.141	−1.487
**Sex** (female/male)	9/33	6/15	3/18	0.111	–
**Physical activity** (min)	332.4 ± 139.5	358.6 ± 159.7	306.2 ± 113.7	0.083	1.753
**Training frequency** (twice/≥three times per week)	5/37	3/18	2/19	0.500	–
**Baseline WBLT** (cm)	13.4 ± 2.6	13.0 ± 2.6	13.8 ± 2.5	0.122	−1.561
**Baseline Y-Balance** (%)					
*Anterior*	64.7 ± 5.4	63.2 ± 6.1	66.3 ± 4.1	0.007	−2.779
*Posteromedial*	105.9 ± 7.9	103.5 ± 6.9	108.3 ± 8.2	0.005	−2.902
*Posterolateral*	100.9 ± 8.6	97.3 ± 8.3	104.5 ± 7.3	<0.001	−4.275
*Composite score*	90.5 ± 6.1	88.0 ± 5.8	93.1 ± 5.4	<0.001	−4.104

*Data are presented as raw total or group mean ± SD*.

a *T-tests and Chi-squared tests were conducted to compare the means of the continuous and categorical variables between the two groups*.

**Figure 4:**
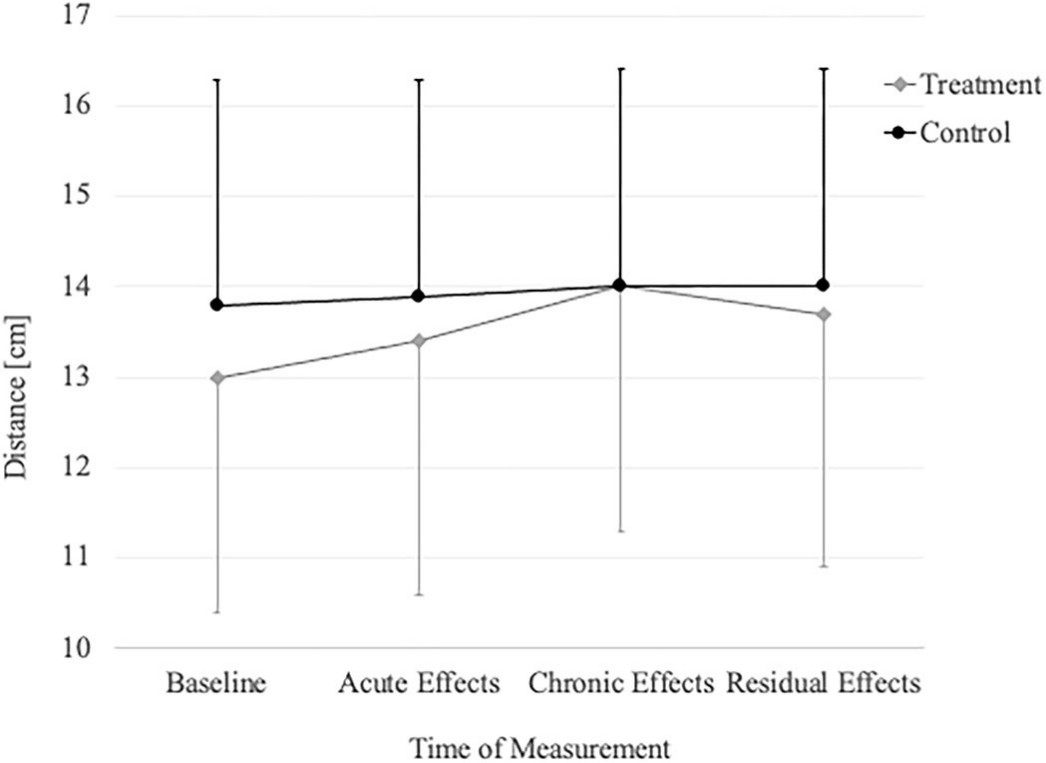
Change in WBLT over time in the treatment group and controls.

**Figure 5:**
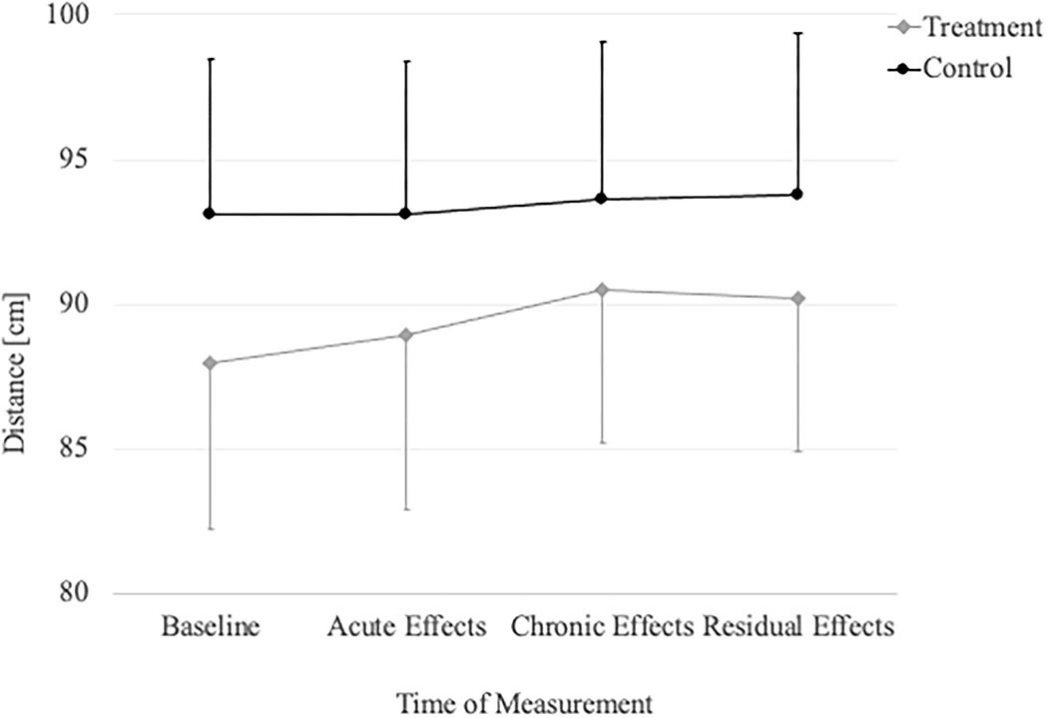
Change in YBT composite score over time in the treatment group and controls.

The repeated measures ANOVA (Table 2) revealed significant time and group by time interaction effects in the WBLT (*p* < 0.001) and the anterior (*p* = 0.021), posterolateral (*p* = 0.003) reach and the composite score (*p* = 0.002) of the Y-Balance test (Figures 4, 5). Only the posteromedial reach (*p* = 0.078) of the Y-Balance test showed no significant effects.

**Table 2:** Repeated measures ANOVA for time and group by time interaction effect.

	**Time effect**			**Group by time effect**		
	***P*-value**	***F***	**Cohen's *d***	***P*-value**	***F***	**Cohen's *d***
**WBLT**	<0.001	27.709	1.164	<0.001	15.814	0.880
**Y-Balance**						
*Anterior*	0.030	3.446	0.408	0.021	3.792	0.430
*Posteromedial*	0.038	3.009	0.381	0.078	2.393	0.335
*Posterolateral*	<0.001	25.981	1.127	0.003	5.312	0.510
*Composite score*	<0.001	18.619	0.953	0.002	5.601	0.523

The results of the *post-hoc* multivariate ANOVA are displayed in Table 3. Significant acute effects were determined for the WBLT (*p* = 0.009) and the posteromedial direction of the Y-Balance test (*p* = 0.049) but not for the anterior (*p* = 0.639) and posterolateral (*p* = 0.317) direction and the composite score (*p* = 0.106). The chronic effects of both WBLT (*p* = 0.007) and all Y-Balance measures (*p* < 0.05) demonstrated statistical significance. The residual effects were significant in the WBLT (*p* < 0.001) and all Y-Balance measures (*p* < 0.05) excepting the posteromedial (*p* = 0.459) reach.

**Table 3:** Mean and statistics of acute, chronic, and residual effects.

	**Mean**		**Mean change after pre-test**		**ANOVA statistics**	
	**Treatment**	**Controls**	**Treatment**	**Controls**	***P*-value**	***F***
**Acute effects** (post I)						
WBLT (cm)	13.4 ± 2.8	13.9 ± 2.4	0.45 ± 0.63	0.11 ± 0.55	0.009	7.158
Y-Balance (%)						
*Anterior*	63.5 ± 5.8	67.0 ± 5.6	0.31 ± 2.94	0.67 ± 3.85	0.639	0.222
*Posteromedial*	104.0 ± 7.0	107.1 ± 7.2	0.49 ± 3.48	−1.25 ± 4.46	0.049	4.008
*Posterolateral*	99.2 ± 7.9	105.6 ± 7.8	1.94 ± 3.77	1.04 ± 4.32	0.317	1.012
*Composite score*	88.9 ± 6.0	93.1 ± 5.3	0.86 ± 2.18	0.00 ± 2.60	0.106	2.675
**Chronic effects** (post II)						
WBLT (cm)	14.0 ± 2.7	14.0 ± 2.4	1.0 ± 0.76	0.14 ± 0.52	<0.001	36.520
Y-Balance (%)						
*Anterior*	64.8 ± 5.6	66.6 ± 4.2	1.62 ± 2.32	0.25 ± 2.23	0.007	7.556
*Posteromedial*	105.6 ± 6.5	107.9 ± 7.9	2.28 ± 3.50	0.22 ± 3.9	0.013	6.389
*Posterolateral*	102.4 ± 7.0	106.6 ± 7.8	5.10 ± 4.69	2.06 ± 4.09	0.003	9.375
*Composite score*	90.5 ± 5.3	93.6 ± 5.5	2.86 ± 2.61	0.71 ± 2.87	0.001	12.915
**Residual effects** (post III)						
WBLT (cm)	13.7 ± 2.8	14.0 ± 2.4	0.75 ± 0.82	0.12 ± 0.57	<0.001	16.708
Y-Balance (%)						
*Anterior*	64.4 ± 5.7	66.5 ± 4.5	1.23 ± 2.3	0.14 ± 2.50	0.042	4.248
*Posteromedial*	104.4 ± 6.3	108.5 ± 7.8	0.93 ± 4.62	0.12 ± 5.30	0.459	0.554
*Posterolateral*	101.9 ± 7.2	106.3 ± 8.0	4.59 ± 5.72	1.75 ± 3.96	0.010	7.003
*Composite score*	90.2 ± 5.3	93.8 ± 5.6	2.21 ± 3.07	0.69 ± 2.92	0.023	5.409

*Data are presented as group mean ± SD*.

## Discussion

The primary aim of this investigation was to examine whether roller-massaging the calf muscles for a 2-week period would produce chronic and residual effects on ankle dorsiflexion ROM and dynamic balance, with the results indicating significant chronic improvements in both outcome measures. With the exception of posteromedial reach, all improvements remained significantly above baseline after 7 days of detraining. The secondary aim of this study was to explore whether roller-massaging the calf muscles for three sets of 60 s would lead to acute effects on ankle flexibility and dynamic postural control. Significant immediate improvements were observed in ROM and the posteromedial reach of the Y-Balance test. No other Y-Balance measures demonstrated significant changes.

### Ankle Dorsiflexion ROM

Following the 2-week intervention, a significant increase in ankle dorsiflexion ROM was observed. As the retention assessment was scheduled 1 day after the last foam rolling session, it can be concluded that these changes were actual training effects and not acute effects. The observed chronic changes in the treatment group are slightly below the reported recommendations for minimal detectable change of the WBLT (Powden et al., 2015) but were significantly different from the changes in controls. We still consider the change practically relevant since a comparable absolute difference in WBLT was reported between young adults with and without chronic ankle instability (John et al., 2019). Our result adds valuable evidence to the limited existing research investigating the long-term effectiveness of foam rolling on ROM. Whereas, Smith et al. (2019) and Kiyono et al. (2020) also reported significant chronic improvements in ankle dorsiflexion ROM, Aune et al. (2019) reported none. A likely explanation for the discrepancy may be the selected sample in Aune et al. (2019)'s study, consisting exclusively of elite athletes (top-division soccer players). This suggests that foam rolling alone may not provide the same effects for long-term ROM changes in highly trained athletes compared to recreationally active individuals. However, this remains to be thoroughly examined.

Our observations are in agreement with Smith et al. (2019) and Kiyono et al. (2020), despite distinct differences in foam rolling protocols between studies. The participants in Smith et al. (2019) study completed 12 foam rolling sessions which were evenly spread over a 6-week period and consisted of three sets of 30 s. Similarly, Kiyono et al. (2020) instructed their participants to foam roll three times a week for three sets of 30 s spanning a 5-week period. The intervention of the present study, by contrast, comprised a 2-week period with six sessions per week and three sets of 60 s. Above all, the comparison of these protocols underlines that foam rolling can induce chronic ROM changes within a relatively short amount of time. Whether this was due to the higher training frequency and volume cannot be ascertained. However, since no clear dose–response relationship exists regarding acute ROM responses to foam rolling (de Souza et al., 2019; Behm et al., 2020), it may be speculated that this relationship is also absent from long-term ROM changes. Future studies on the current topic should therefore seek to clarify the relation between dose and response by incorporating two or more different foam rolling protocols within one trial.

Nine days post-intervention, the chronic ROM changes were still significant, which supports existing reports of ROM being retained after 1 week of detraining (Smith et al., 2019). Although it is currently unclear how long these changes in ROM following foam rolling are preserved, hamstring flexibility improvements return to baseline 4 weeks after a 6-week stretching protocol (Willy et al., 2001), and improvements in ankle dorsiflexion are no longer present 5 weeks following a 5-week stretching program (Nakamura et al., 2021a), which may indicate similar detraining timelines for foam rolling interventions. Future studies should seek to clarify this.

Apart from chronic and residual effects, the present study revealed significant acute effects on ROM following three sets of 60 s of foam rolling. The outcome is well-aligned with earlier studies investigating acute responses to foam rolling on ankle dorsiflexion ROM (Halperin et al., 2014; Kelly and Beardsley, 2016; de Souza et al., 2019; Yoshimura et al., 2021). Although between-study heterogeneity makes it difficult to compare the results, it is noteworthy that Yoshimura et al. (2021) found a remarkably higher ROM increase of 22% despite using the same rolling duration (three sets of 60 s). The inconsistency between dose and response is further emphasized by de Souza et al. (2019) who reported an immediate 11% ROM improvement regardless of protocol length (two sets of 30/60 s). Training volume alone therefore may not be a major determinant of the treatment outcome, and instead other variables such as intra and inter individual differences may be more predictive of ROM improvements following foam rolling interventions and should be investigated further in order to optimize protocol recommendations.

There is some debate regarding whether morphological or neural adaptations are primarily responsible for the positive treatment effects of foam rolling (Behm and Wilke, 2019). Wilke et al. (2019) observed reduced tissue stiffness in the anterior thigh immediately after one bout of foam rolling, and recent evidence indicates that changes in gastrocnemius stiffness accompany changes in ankle ROM (Chang et al., 2021). However, there is contradictory evidence from Baumgart et al. (2019), who reported decreased stiffness in the quadriceps but not the calves despite identical foam rolling protocols. In addition, other works examining the gastrocnemius muscle reported that foam rolling caused neither acute (Nakamura et al., 2021b) nor chronic (Kiyono et al., 2020) reductions in tissue stiffness. Based on these inconsistent results, it may be argued that the primary drivers of the ROM enhancement in the current study are neural. Evidence to support this notion is provided by Aboodarda et al. (2015) and Cheatham and Kolber (2018) who reported a reduced pain perception of both the massaged and non-massaged limb after foam rolling. It can be inferred from these findings that foam roll training can affect the central nervous system and its response to pain. Further, the increased threshold of pain might be linked to an increased stretch tolerance, which is considered to be one of the main influencing factors behind ROM gains in the stretch-related literature (Magnusson et al., 1996; see also Blazevich et al., 2014; Freitas et al., 2018). Even though stretching and foam rolling are different in many respects, they can both provide an uncomfortable or painful stimulus capable of altering the neuronal and sensorimotor responses (Behm and Wilke, 2019). Moreover, recent evidence by Kiyono et al. (2020) and Nakamura et al. (2021b) shows that changes in dorsiflexion ROM are significantly correlated with passive torque (i.e., stretch tolerance) but not muscle stiffness.

### Dynamic Balance

The present study provides the first evidence for chronic changes in dynamic balance induced by foam rolling. Although Y-Balance test scores dropped after 7 days of detraining, they remained above baseline. Therefore, it appears that improvements in chronic ankle ROM are accompanied by improvements in balance performance following a foam rolling intervention. However, although statistically significant, the reported changes in all reaching directions were below the minimal detectable change reported by Powden et al. (2019).

Since ankle dorsiflexion ROM is most strongly correlated with the anterior reach performance (Hoch et al., 2011; Basnett et al., 2013), it may be considered surprising that the magnitudes of the chronic and residual effects of the anterior reach were exceeded by the posterolateral reach and nearly equaled by the posteromedial reach. To explain this outcome, the following two points should be considered: first, hip and knee flexion are strong predictors of posterolateral and posteromedial reach performance (Robinson and Gribble, 2008), and second, altered hip and knee kinematics strongly correlate with decreased ankle dorsiflexion ROM (Rabin et al., 2016). Therefore, it is plausible that increased ankle flexibility led to improved hip and knee movement patterns which, in turn, enabled greater posterior reach distances.

Our findings are not consistent with those of Junker and Stöggl (2019) who found no positive developments in dynamic balance after 8 weeks of lower body foam rolling. Their training program, conducted twice a week, targeted the calves, quadriceps, hamstrings, IT-band, and glutes. Even though Junker and Stöggl (2019) did not assess ankle dorsiflexion, an increase in ROM most probably occurred, considering that the calf muscle protocol was comparable to the ones used in Smith et al. (2019) and Kiyono et al. (2020) study. It thus seems unlikely that the contradictory results between the present study and Junker and Stöggl (2019) are related to differences in foam rolling protocols. Instead, it could be assumed that different assessment methods might have contributed to the controverse findings, as Junker and Stöggl (2019) reported considerably greater reach distances in the composite score.

Finally, of all Y-Balance measures, only the posteromedial reach showed significant acute improvements. However, the magnitude of the effect was small and thus of little practical relevance. Considering the acute dorsiflexion ROM gains and the chronic balance improvements, the result was unexpected. This inconsistency may be attributed to one of the following two reasons. First, depending on roller–massager position, the quadriceps of the massaged limb was always engaged either concentrically or eccentrically. This movement might have provided an unusual stimulus capable of inducing muscle fatigue in the quadriceps. Since muscle fatigue protocols applied to the quadriceps can significantly reduce Y-Balance test performance (Fatahi et al., 2016), the absence of immediate balance improvements might have been due to a decrease in maximal force production. Second, Lee et al. (2018) found that vibration rolling and non-vibration rolling of the quadriceps and hamstrings (three sets of 30 s) led to immediate improvements in dynamic postural control. Bearing this in mind, it might be more recommendable to foam roll the quadriceps and hamstrings instead of the gastrocnemius muscle to acutely affect dynamic balance.

### Limitations

Several limitations need to be considered when interpreting the findings of the current study. Firstly, all assessments were performed under non-blinded circumstances, which increases the risk of bias. Second, after familiarization with the foam rolling technique, the correct execution was only supervised on *Day 1*. The following 12 training sessions of the 2-week protocol were home-based and therefore not monitored. Third, all participants were instructed to apply as much pressure as possible with the roller–massager, which invites a high degree of variability. Fourth, the roller–massager was made from wood, which may limit the findings to the use of wooden foam rolling devices. Fifth, all participants agreed to maintain their normal exercise routine, and differences in this exercise routine were not accounted for in our analysis. Sixth, the subjects were aged between 18 and 35 and recreationally active, and so the transfer of our findings to other populations cannot be ascertained. Another limitation is the lack of clear definition for acute, chronic, and residual effects in the literature. In our study, we used the second test immediately after the first RM for acute effects, the 14 days intervention period for chronic effects, and the subsequent 7 days without intervention for residual effects. Finally, the time points for the three categories (acute, chronic, residual) in our study may differ compared to other studies due to the lack of specific recommendations in the literature.

## Conclusion

This study demonstrated that a 2-week period of regular calf muscle rolling chronically improved ankle dorsiflexion ROM and dynamic balance, and that these positive improvements in functional performance were retained after 7 days of detraining in all but one of the post III measurements, underlining the sustainability of foam rolling effects. Further, foam rolling acutely improved ROM despite mixed findings on immediate balance performance.

## Practical Relevance

In practical terms, these findings support calf muscle roller-massaging for (1) warm-up if acute increases in ankle dorsiflexion ROM are required and (2) regular use to produce long-term improvements in ankle dorsiflexion ROM and dynamic balance. The findings also indicate that improvements in ROM and balance persist for longer periods (at least 1–2 weeks) in the absence of foam rolling.

## Data Availability Statement

The original contributions presented in the study are included in the article, further inquiries can be directed to the corresponding author.

## Ethics Statement

The studies involving human participants were reviewed and approved by the Ethics Committee of the Faculty of Social and Behavioural Sciences of the Friedrich Schiller University Jena, Germany. The participants provided their written informed consent to participate in this study. Written informed consent was obtained from the individual for the publication of the potentially identifiable image included in this article.

## Author Contributions

TS, JM, and AZ were fully involved in the study and preparation of the manuscript. All authors have read and concur with the content in the final manuscript.

## Conflict of Interest

The authors declare that the research was conducted in the absence of any commercial or financial relationships that could be construed as a potential conflict of interest.

## Publisher's Note

All claims expressed in this article are solely those of the authors and do not necessarily represent those of their affiliated organizations, or those of the publisher, the editors and the reviewers. Any product that may be evaluated in this article, or claim that may be made by its manufacturer, is not guaranteed or endorsed by the publisher.
